# Developing Forensic Mental Healthcare in Kosovo

**DOI:** 10.3389/fpubh.2014.00026

**Published:** 2014-04-07

**Authors:** Hans Joachim Salize, Juha Lavikainen, Allan Seppänen, Milazim Gjocaj

**Affiliations:** ^1^Central Institute of Mental Health, Medical Faculty Mannheim/Heidelberg University, Mannheim, Germany; ^2^Whiteray & Delving Ltd., Helsinki, Finland; ^3^Vanha Vaasa Hospital, Vaasa, Finland; ^4^Ministry of Health of Republic of Kosovo, Pristina, Kosovo

**Keywords:** forensic psychiatry, capacity building, international collaboration, mentally ill offenders, mental health care, public mental health, psychiatric training

## Abstract

In many economically struggling societies, forensic psychiatry is still in its initial developmental stages and thus forensic patients pose an ongoing challenge for the healthcare and juridical systems. In this article, we present the various issues and problems that arose when establishing the first forensic psychiatric institute in Kosovo – a country whose population has constantly been reported as suffering from a high psychiatric morbidity due to long-lasting traumatic experiences during the war of 1999. The implementation of a new forensic psychiatric institute in the developing mental healthcare system of Kosovo, still characterized by considerable shortages, required substantial effort on various levels. On the policy and financial level, it was made possible by a clear intent and coordinated commitment of all responsible national stakeholders and authorities, such as the Ministries of Health and Justice, and by the financial contribution of the European Commission. Most decisive in terms of the success of the project was capacity building in human resources, i.e., the recruitment and training of motivated staff. Training included essential clinical and theoretical issues as well as clearly defined standard operation procedures, guidelines, and checklists to aid daily routine work and the management of challenging situations.

## Introduction

Most industrialized countries have established a structure for placing and treating mentally ill offenders in specialized facilities and services, either as a specialized sector within the healthcare system or more or less integrated into the overall scheme of psychiatric care. In some countries, forensic psychiatry is organized as a part of the prison mental health care services. However, concepts, models, specialized services, and quality of care differ widely across Europe and worldwide (1). In many threshold countries or economically struggling societies, forensic psychiatry is still in its initial stages and poses an ongoing challenge for the healthcare and juridical systems and society in general. This is the case in Kosovo, where a process for establishing a new specialized service for mentally ill offenders – the first forensic psychiatric institute of the country – was initiated and included in the Kosovo mental health strategy for 2008–2013. The purpose of this paper is to describe the preparation and implementation processes for that service in Kosovo and the issues and problems to be addressed when establishing a new forensic psychiatric institute in an economically struggling society characterized by a limited mental health care infrastructure and rudimentary experience in forensic psychiatry.

## Materials and Methods

### Starting point

#### Demographics

Kosovo is a land-locked country in the Western Balkans with a territory of ca. 10,000 km^2^ and a population of ca. 1.8–2 million. More than 90% of the population is Albanian, whereas the Serb minority is ca. 5–7%. Following roughly a decade under Serbian regime that culminated in the war at the end of the 1990s, on June 1, 1999, Kosovo was placed under UN administration, which established the United Nations Interim Administration Mission in Kosovo (UNMIK). Kosovo declared its independence on February 17, 2008 (2). EULEX, the European Union Rule of Law Mission, whose major aim is to assist and support the Kosovo authorities in the police, judiciary, and customs services, has been operating in Kosovo since 2009 (2). Still suffering from repercussions of the war, Kosovo’s economy is small and the population is poor with an annual per capita GDP of ca. 1.500 €. The economy is characterized by relatively low foreign investments and a high level of inflows, which include considerable donors and remittances from people from Kosovo working abroad (2). The infrastructure has witnessed some progress, but is still insufficient with regular power cuts and a high level of pollution from the country’s outdated coal-fired power plant (2). The health care system is underdeveloped with some basic national health services, both hospital and outpatient based, run by the ministry of health and a private sector whose actual size is hard to estimate. A mandatory health insurance scheme is not implemented.

#### Prevalence of mental ill health

The prevalence of mental ill health in Kosovo has constantly been reported as very high. In 2005, the Kosovo Rehabilitation Centre for Torture Victims (KRCT) found 27.7% of the population having substantial psychiatric morbidity, indicated by a General Health Questionnaire-28 score of 12 and higher (3). As a long-term consequence of the Kosovo war, post-traumatic stress disorder (PTSD), depression, and emotional distress remained high among the population, with 22% prevalence of PTSD symptoms, 41.8% prevalence for depression (HSCL-20 score), and 43.1% prevalence for emotional distress. These scores from 2005 had only slightly decreased from those in the year 2000 (3). Similarly, 64.9% of the population was reported to have had traumatic experiences during the war, resulting in ca. 200,000–400,000 traumatized persons in addition to the baseline figure of 200,000–300,000 mentally disordered persons that are to be expected in the Kosovo general population (4). More recent studies suggest that the war-related prevalence of mental illness is only slowly decreasing (5–7).

These lasting psychological consequences of the war, combined with a high rate of uncontrolled weapons in a country with legal structures still under development, would suggest an above average rate of crimes committed under the influence of a mental disorder. However, neither the prevalence nor incidence, nor indeed any reliable indicator concerning mentally ill offenders in Kosovo, are currently available.

#### Mental healthcare and forensic psychiatry in Kosovo

In 2013, mental health care as provided by governmental budgets includes general psychiatric hospital services located in the capital of Pristina and in the major regional municipalities of Kosovo (Peja, Prizren, Gjakova, and Gjilan). With the exemption of the Neuropsychiatry Clinic of the University Clinical Centre of Kosovo in Pristina, which provides the majority of psychiatric inpatient capacities of Kosovo (88 beds), regional psychiatric wards are equipped with 10–25 beds on average. In 2012, all in all 166 psychiatric beds were provided for Kosovo at large. This is a psychiatric bed rate of ca. 8.3 per 100,000 population, which is roughly 10 times less than in Central European or Scandinavian countries. In municipalities with a psychiatric ward, integration houses and community mental health centers have been established, too, providing long-term rehabilitative care for the chronically mentally ill. Additionally, the Shtime Rehabilitation Center, a centralized service providing long-term care for mentally disordered or intellectually disabled people from all over Kosovo, has 58 beds for male and female patients available. Whereas existing mental health care services seem to be more or less sufficiently staffed, the treatment options are limited due to a serious lack of essential resources, such as second-generation antipsychotic drugs or the coverage of an appropriate community mental health care network all over the country.

#### Forensic psychiatric services in Kosovo

Until 2013, the only available service for mentally ill offenders in Kosovo was a three room unit with 11 beds in the Neuropsychiatry Clinic of the University Clinical Centre in the capital. The unit was mainly used for forensic psychiatric assessment; long-term inpatient treatment for mentally disordered offenders was rare (8). The majority of offenders suffering from a mental disorder are detained in the Medical Ward of Dubrava Prison, which is the country’s largest prison with ca. 1.600 inmates. The medical prison ward in Dubrava has 16 beds designated for acute treatment of psychiatric cases, but it suffers from the same supply-shortages as do the regional psychiatric wards. The prison ward is poorly staffed with nursing and other specialist mental health care staff. Patients with serious somatic and mental disorders may share the same room. Visits by psychiatrists are scheduled on a weekly basis. For less severely mentally ill inmates, some additional places are provided in a rehabilitatively oriented prison ward.

The existing facilities have been seriously criticized by the Judicial Inspectorate of the Kosovo Judicial Council and the Committee for Prevention of Torture (9). Additionally, EULEX, operating under the United Nations Security Council Resolution 1244 (10), has stated the lack of adequate detention facilities for offenders with diminished mental capacity in its European Commission (EC) 2010 progress report.

#### Legal provision for placing and treating mentally ill offenders

One of the most crucial steps in laying down the juridical preconditions for placing and treating mentally ill offenders in Kosovo was taken in 2004 when the UNMIK-Regulation no. 2004/32 on Criminal Proceedings involving Perpetrators with a Mental Disorder came into effect (11). The regulation defines trial procedures for offenders suspected to be suffering from a mental disorder, including their placement and treatment, in line with international juridical and medical standards. The Criminal Code No 04/L-082 of the Republic of Kosovo additionally regulates that, if an offense was committed by a person considered as mentally incompetent or having diminished mental capacities, the court has to take the mental condition of the offender into consideration when deciding the type and duration of a sanction or measure of mandatory treatment (12).

### Establishment of Kosovo Forensic Psychiatry Institute

#### Policy process

A general mental health reform was initiated in Kosovo already in 2001 (13). In light of the aforementioned weaknesses in the forensic psychiatric services, the Kosovo Strategy on Mental Health 2008–2013 provided that a new Kosovo Forensic Psychiatric Institute (KFPI) should be built and implemented as a service for assessing and treating persons having committed a crime under the influence of a mental disorder. The EC, implementing its enlargement perspective, had been actively pursuing to develop the rule of law in Kosovo through its own instruments, such as the instrument for pre-accession assistance (IPA). As a part of this process, the EC has a substantial impact on the development of health care in the country, and it decided to support the Ministry of Health and the Ministry of Justice of Kosovo in establishing a new specialized service for mentally ill offenders under the Annual Program for Kosovo under IPA 2010 component 1 (2). In November 2009, a memorandum of understanding was signed between the Ministry of Justice and the Ministry of Health, which agreed upon the implementation of the KFPI. The EC, through their annual Program for Kosovo, provided support in establishing the KFPI. The expenses for 30 future staff members were included into the Midterm Expenditure Framework of the Government of Kosovo. The recently revised mental health strategy of Kosovo (2014–2020) depicts the forensic psychiatry services as an integral part of the whole mental health care service provision.

#### Developmental process as a project

After the tendering process, which was initiated and finalized in late 2011, the Finnish National Institute for Health and Welfare (THL) was selected to carry out the project “Establishment of Kosovo Forensic Psychiatry Institute (KFPI).” The project was funded by the EC, managed by the European Union Office in Kosovo and implemented by THL. The project was carried out together with the support of local beneficiaries from the Ministry of Health, the Ministry of Justice, the Kosovo Judicial Council, and the Psychiatric Clinic of the University Clinical Centre of Kosovo. A steering committee was established that regularly reviewed the progress reports and monitored the advancement of the project along the preset objectives.

The goal was to establish adequate facilities to conduct forensic psychiatric examinations for the courts and to provide mandatory treatment for mentally ill offenders. Hence, the quality of treatment would be significantly improved by both the provision of appropriate facilities and by a professional multidisciplinary staff specifically trained to carry out high quality care in this new environment. In particular, the processes included the development of appropriate working conditions and collaborative structures of the KFPI with the network of existing juridical and psychiatric services in Kosovo and the training of future KFPI-staff members and other stakeholders according to internationally accepted standards of forensic psychiatry. The construction works of the building for the KFPI, which was supposed to be opened by fall 2013 (eventually delayed until spring 2014), was supported in a parallel process by the EU through a separate service contract.

#### Methods

To work on and implement effective and sustainable clinical, organizational, and management structures and activities for the KFPI, an expert team was set up, which included a team leader from Finland, a local office manager and one short-term expert from Finland and Germany each. The project team combined medical, forensic psychiatric, psychological, sociological, and epidemiological expertise and received strong support and supervision from a steering group and the European Union Office in Kosovo (14). The project started in March 2012 and lasted until September 18, 2013. The project included the following tasks:

- review of the current state of service provision for mentally ill offenders in Kosovo,
- definition of the main needs, role, and responsibility of the new forensic psychiatric institute,
- definition of job descriptions,
- definition of recruitment policies and support in developing recruitment documents,
- definition of standard operating procedures (SOPs) for all aspects of forensic service provision, addressing also the interfaces and collaboration with the Kosovo juridical system and the general psychiatric services,
- training of future staff of the institute and stakeholders.

## Results

The review of the state of Kosovo’s mental healthcare and forensic psychiatric care as part of the initial task is summarized in the Introduction and Starting point sections above. For the definition of jobs, roles, and responsibilities training groups were formed, which included physicians, psychologists, nurses, and social workers of the future institute as well as judges from regional courts and representatives of general psychiatric services and of the Ministries of Health and Justice. Key NGOs were heard and consulted in a specific workshop at the very beginning of the project.

The training of the future staff and other stakeholders was provided in a stepwise format and was split into several modules. A basic training, which addressed general issues relevant to forensic psychiatry took place on 9 days in October and November 2012 and an advanced training attended only by the future staff members and addressing more detailed treatment and care issues followed on 4 days in March and April 2013. For the contents of the basic training modules, see Table 1.

**Table 1:** **Modules for basic training of stakeholders and staff of the Kosovo Forensic Psychiatric Institute**.

Module	Content
Patients’ and human rights aspects in forensic psychiatry	Resolutions, charters, declarations, agencies
	Role of European Union and United Nations
	Specific rights, defense council, long-lasting consequences, stigma
Forensic legislation and practice – European overview and standards	European models of forensic legislation and care
	Compulsory treatment in general psychiatry
	Prison mental healthcare
Legal and organizational preconditions in Kosovo	Current situation and needs
	Changes by establishing KFPI
	Models of best practice
Forensic psychiatric assessment	Legal prerequisites and scope of legislation
	Assessment methods
	Professional background, quality standards
Forensic psychiatric treatment	Methods, special programs
	Security aspects
	Outpatient forensic care
Discharge procedures and aftercare	Aftercare provision, link to general psychiatry
	Collaboration with social services
Communication and responsibilities between medical and legal professions	Communication skills, standards, platforms
	Feedback and reporting procedures
	Joint conferences and training
Management of violence and risks	Risk assessment
	Training of staff members
	Collaboration with police
Future perspective of forensic psychiatry in Kosovo	Vision
	Curricula for forensic psychiatric training

Whereas the list of the training modules in Table 1 gives a rough overview of the areas of essential knowledge that were transferred to the local professionals and stakeholders, the standard operation procedures (SOPs) give guidelines and checklists available to staff members for daily routine work and the management of challenging situations in a forensic psychiatric ward. An SOP in that context is a written set of instructions that someone should follow to complete a job safely, with no adverse effect on personal health or the environment, and in a way that maximizes operational efficiency (15). The SOPs for the KFPI are divided into three major parts addressing admission, general security issues, and limitations on the right of self-determination. They cover and regulate such issues as searching a patient or his/her personal property, involuntary treatment of a patient, maintaining patient-safety during isolating or tying and limiting the right to leave. As an example, Table 2 shows the areas and issues detailed in the nurses’ checklist that serves as a guideline during the admission process of a new patient. It exemplifies the areas to be covered when improving the legal knowledge and technical skills that are essential for staff working in such a challenging environment.

**Table 2:** **Nurses checklist for new entries as part of the SOPs for the Kosovo Forensic Psychiatric Institute (abbreviated)**.

Notification procedures on arrival
Body search and frisking if ordered by a physician
Recording of patient’s identifying characteristics
Checking, taking possession, and storing personal property
Consent to disclosure of information to outside KFPI
Information about patient’s rights
Information about video or other surveillance in the wards
Introduction to wards
Patient medication according to physician’s orders
Medical examinations according to physician’s orders
Regulating visits
Treatment plan

**Figure 1:**
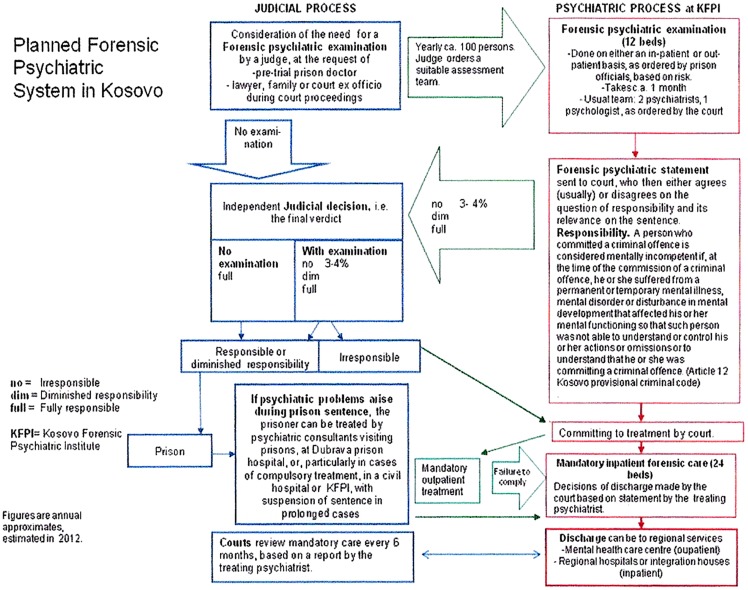
**Forensic psychiatric system in Kosovo**.

As a basis for the training sessions, a chart depicting a general overview of the psychiatric system in Kosovo was drafted. It illustrates the interfaces with the juridical system, the legal provisions, and the pathways for placing and treating mentally ill offenders in the future KFPI (see Figure 1). This chart was an important tool for defining and discussing the roles, tasks, and duties of medical and juridical authorities, KFPI-staff members and the various stakeholders in the forensic psychiatric services. It also was helpful for highlighting the critical period after discharge of a patient from the KFPI, which requires adequate forensic psychiatric aftercare and strong collaborative structures with regional general psychiatric services. Structured communication procedures between these three sectors (forensic psychiatry, courts and judges, general psychiatry) were developed and recommended for implementation.

The capacity building process was officially completed by carrying out a study visit of key KFPI-staff members and decision makers to Finland, where the National Institute for Health and Welfare and Vanha Vaasa forensic psychiatric hospital were visited and workshops and meetings with Finnish experts were held.

## Discussion

The implementation of a new forensic psychiatric institute in a developing mental healthcare system that is relatively unfamiliar with such specific services requires substantial effort and activities on various levels. On the policy level, in the case of Kosovo, the mission was made possible by a clear intent and coordinated commitment of all responsible authorities and by the financial contribution of the EC. Given the specific status of Kosovo, having declared independence only recently and having been supported by UN and EU authorities and programs for more than a decade, international initiative, approval, and financial support was decisive. On that basis, all further steps and activities of both the construction and implementation process of the new forensic psychiatric institute were made possible in a structured and coordinated manner. This process involved national focal points, stakeholders, and professionals from the administrative, governmental, non-governmental, juridical, prison, healthcare, and general psychiatric sectors, which highlights the multi-dimensional or system aspect of the process. The collaboration of each sector was crucial.

The Steering Committee of the project was fully competent and devoted to carry out its supervisory role. A timely and well-prepared independent results oriented monitoring process was carried out while the project was still running. This monitoring was very positive with regard to all project elements.

During the stages of the implementation process described in detail in this paper, there was no evidence of any significant friction between the beneficiaries, at least not visible to the international project group. Quite the contrary, the process turned out to develop synergies in related areas, which in turn were quite beneficial for the actual project. Thus, the implementation of structures and capacities for the new Kosovo Psychiatric Forensic Institute had a substantial impact on the drafting of the Kosovo mental health law, which was under way as a parallel, although independent, process. The new mental health law regulates involuntary treatment of civil committed persons and mentally ill offenders alike and it includes rules for the protection of human and fundamental rights of persons concerned. These rules were partly influenced by the respective chapters and paragraphs of the SOPs of the KFPI (13). Additionally, a new Kosovo mental health strategy for the years 2014–2020 had been prepared simultaneously by the Ministry of Health, in which the key role of the KFPI in the network of mental health care services was highlighted.

Most decisive in terms of the success of the project was capacity building in human resources, i.e., the recruitment and training of motivated staff. The training, as described above, did not only include medical and nursing staff, who are pivotal for the care and quality standards for the new service, but also administrators and closely collaborating representatives from the juridical sector.

Having secured the multi-professional and cross-sectoral character of the process, the implementation of the new Kosovo Forensic Psychiatric Institute (or the National Center for Forensic Psychiatry, as the Institute’s Advisory Board has recently decided to rename the organization) has the potential not only to be functional and highly effective in its actual area – to assess mentally ill offenders in Kosovo and provide adequate care for this high-risk group – but also to be influential to and affect other fields. The new service has the potential to work as a benchmark for other psychiatric services in Kosovo and thus increase the overall quality of mental healthcare in the country. Moreover, a functional KFPI might, at best, act as a role model for similar initiatives in the whole Balkan region, which is suffering from similar shortcomings in forensic psychiatry (16). However, since inclusion of users and families in any development of new mental services is widely regarded as good practice and has for several reasons not been the case in the project described, this might be seen as a limitation and should be included into any replication in other countries.

## Conflict of Interest Statement

The project “Establishment of Kosovo Forensic Psychiatry Institute (Europeaid/131672/C/SER/XK)” described in this article was funded under the Service Contract No. 2012/284-009 between the European Union represented by the European Union Office in Kosovo and the National Institute for Health and Welfare in Finland (THL). Hans Joachim Salize, Juha Lavikainen, and Allan Seppänen worked as paid senior or short-term experts in the Project. During the time covered by the article, Milazim Gjocaj worked for the Ministry of Justice of the Republic of Kosovo, who was a beneficiary of the Project. Any other potential conflict of interest is disclosed.
